# Supplementary material for: Exosome-Transmitted tRF-16-K8J7K1B Promotes Tamoxifen Resistance by Reducing Drug-Induced Cell Apoptosis in Breast Cancer
Source: Cancers (Basel). 2023 Jan 31;15(3):899. doi: 10.3390/cancers15030899 (PMC9913720; doi:10.3390/cancers15030899)
Supplement: Supplementary file 1 [file cancers-15-00899-s001.zip › Table S1.pdf]

Table S1. Clinical characteristic of breast cancer patients

Variables		Number(N=56)	
**Age**			
	<50	30	53.60%
	≥50	26	46.40%
**Tumor size**			
	T1/T2	51	91.10%
	T3/T4	5	8.90%
**Lymph node metastasis**			
	N0	12	21.40%
	N1	24	42.90%
	N2	13	23.20%
	N3	7	12.50%
**Pathological stage**			
	I	4	7.10%
	II	32	57.20%
	III	20	35.70%
**Histological grade**			
	I	3	5.40%
	II	40	71.40%
	III	13	23.20%
**ER status**			
	ER positive	54	96.40%
	ER negative	2	3.60%
**PR status**			
	PR positive	36	64.30%
	PR negative	20	35.70%
**HER2 status**			
	HER2 positive	15	26.80%
	HER2 negative	41	73.20%
**Ki67 status**			
	≤15%	22	39.30%
	>15%	34	60.70%
