# Supplementary material for: Exosome-Transmitted tRF-16-K8J7K1B Promotes Tamoxifen Resistance by Reducing Drug-Induced Cell Apoptosis in Breast Cancer
Source: Cancers (Basel). 2023 Jan 31;15(3):899. doi: 10.3390/cancers15030899 (PMC9913720; doi:10.3390/cancers15030899)
Supplement: Supplementary file 1 [file cancers-15-00899-s001.zip › Table S2.pdf]

Supplementary Table S2: Sequence of primers of tRFs and tiRNAs used for qRT-PCR.

Name	Sequence
tRF-18-HRE9XFD2 RT	GTCGTATCCAGTGCAGGGTCCGAGGTATTCGCACTGGATAC GACTGGTGT
tRF-18-HRE9XFD2 Forward	CGCGCGATCCCACTTCTG
tRF-18-HRE9XFD2 Reverse	AGTGCAGGGTCCGAGGTATT
tRF-17-WSNKP92 RT	GTCGTATCCAGTGCAGGGTCCGAGGTATTCGCACTGGATAC GACTGGAGG
tRF-17-WSNKP92 Forward	GCGCGTCTCGCTGGGG
tRF-17-WSNKP92 Reverse	AGTGCAGGGTCCGAGGTATT
tRF-28-6978WPRLXND RT	GTCGTATCCAGTGCAGGGTCCGAGGTATTCGCACTGGATAC GACGAGAAT
tRF-28-6978WPRLXND Forward	GCTCGTTGGTCTAGGGGTATG
tRF-28-6978WPRLXND Reverse	AGTGCAGGGTCCGAGGTATT
tRF-16-489B3RB RT	GTCGTATCCAGTGCAGGGTCCGAGGTATTCGCACTGGATAC GACTGGAGG
tRF-16-489B3RB Forward	CGCGCGCTCGGTGGAA
tRF-16-489B3RB Reverse	AGTGCAGGGTCCGAGGTATT
tRF-16-K8J7K1B RT	GTCGTATCCAGTGCAGGGTCCGAGGTATTCGCACTGGATAC GACTGGTGG
tRF-16-K8J7K1B Forward	CGCGCGCCCGGCATCT
tRF-16-K8J7K1B Reverse	AGTGCAGGGTCCGAGGTATT
tRF-18-BS68BFD2 RT	GTCGTATCCAGTGCAGGGTCCGAGGTATTCGCACTGGATAC GACTGGTGT
tRF-18-BS68BFD2 Forward	CGCGAACCGGGCGGAA
tRF-18-BS68BFD2 Reverse	AGTGCAGGGTCCGAGGTATT
tRF-18-HR0VX6D2 RT	GTCGTATCCAGTGCAGGGTCCGAGGTATTCGCACTGGATAC GACTGGTGG
tRF-18-HR0VX6D2 Forward	CGCGATCCCACCGCTG
tRF-18-HR0VX6D2 Reverse	AGTGCAGGGTCCGAGGTATT
tRF-32-P4R8YP9L RT	GTCGTATCCAGTGCAGGGTCCGAGGTATTCGCACTGGATAC GACAGGCGA
tRF-32-P4R8YP9L Forward	TGGGTGGTTCAGTGGTAGAATTC
tRF-32-P4R8YP9L Reverse	AGTGCAGGGTCCGAGGTATT
tRF-32-PNR8YP9L RT	GTCGTATCCAGTGCAGGGTCCGAGGTATTCGCACTGGATAC GACAGGCGA
tRF-32-PNR8YP9L Forward	TTGGTGGTTCAGTGGTAGAATTC
tRF-32-PNR8YP9L Reverse	AGTGCAGGGTCCGAGGTATT
tRF-30-R9JP9P9NH5HY RT	GTCGTATCCAGTGCAGGGTCCGAGGTATTCGCACTGGATAC GACCAGATG
tRF-30-R9JP9P9NH5HY Forward	GGGTTCCATAGTGTAGTGGTTATCA

tRF-30-R9JP9P9NH5HY Reverse	AGTGCAGGGTCCGAGGTATT
U6 RT	AACGCTTCACGAATTTGCGT
U6 Forward	CTCGCTTCGGCAGCACA
U6 Reverse	AACGCTTCACGAATTTGCGT
TNFSF10 forward	GCTGAAGCAGATGCAGGACAAG
TNFSF10 reverse	GCTGACGGAGTTGCCACTTGAC
GAPDH Forward	ATCAAGTGGGGCGATGCTG
GAPDH Reverse	ACCCATGACGAACATGGGG
