# Supplementary material for: Exosome-Transmitted tRF-16-K8J7K1B Promotes Tamoxifen Resistance by Reducing Drug-Induced Cell Apoptosis in Breast Cancer
Source: Cancers (Basel). 2023 Jan 31;15(3):899. doi: 10.3390/cancers15030899 (PMC9913720; doi:10.3390/cancers15030899)
Supplement: Supplementary file 1 [file cancers-15-00899-s001.zip › Table S3.pdf]

Supplementary Table S3: Antibody

Antigens	Manufacturer	Application
CD9	Affinity，AF5139	1:1000 for WB
CD63	Affinity，AF5117	1:1000 for WB
TSG101	Proteintech，14497-1-AP	1:1000 for WB
Alix	Proteintech，12422-1-AP	1:1000 for WB
TRAIL	Proteintech，27064-1-AP	1:1000 for WB, 1:500 for IHC
Cleved Caspase 3	Affinity，AF7022	1:1000 for WB, 1:500 for IHC
Cleved PARP	Affinity，AF7023	1:1000 for WB, 1:500 for IHC
β-Actin	Proteintech，66009-1-Ig	1:1000 for WB
