# Supplementary figures and images for: Exosome-Transmitted tRF-16-K8J7K1B Promotes Tamoxifen Resistance by Reducing Drug-Induced Cell Apoptosis in Breast Cancer
Source: Cancers (Basel). 2023 Jan 31;15(3):899. doi: 10.3390/cancers15030899 (PMC9913720; doi:10.3390/cancers15030899)

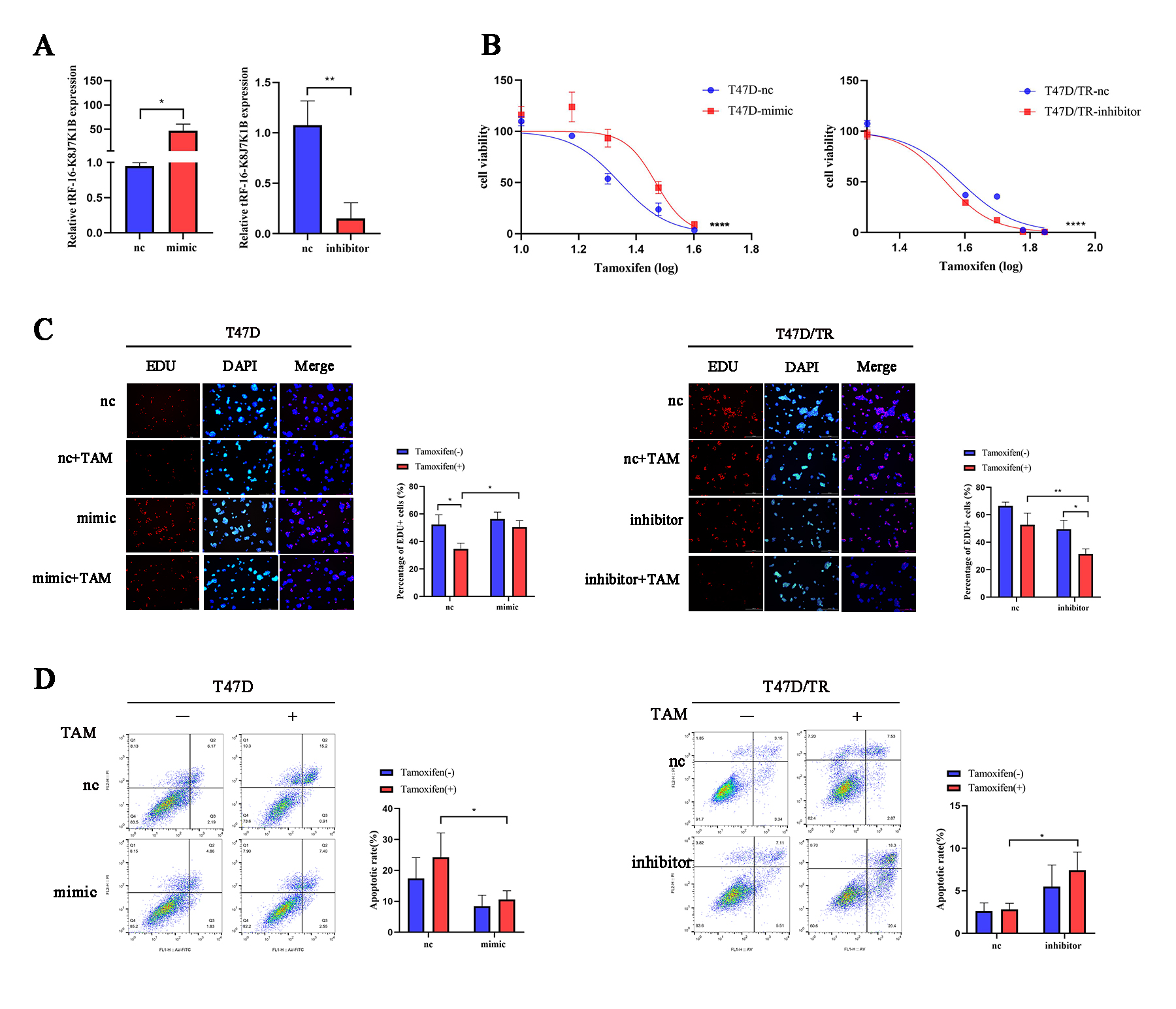

Supplement: Supplementary file 1 [file cancers-15-00899-s001.zip › Figure S1.jpg]

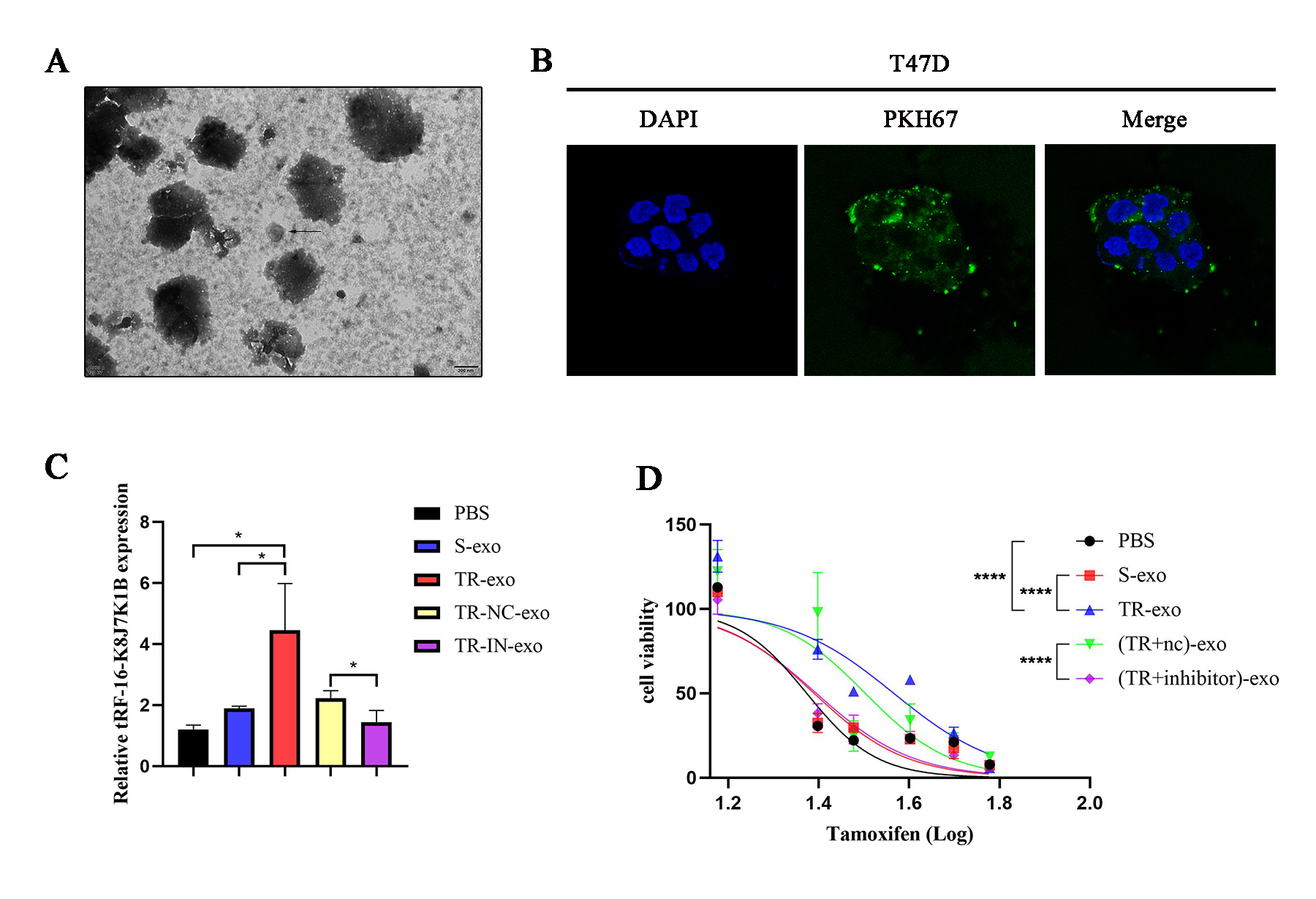

Supplement: Supplementary file 1 [file cancers-15-00899-s001.zip › Figure S2.jpg]

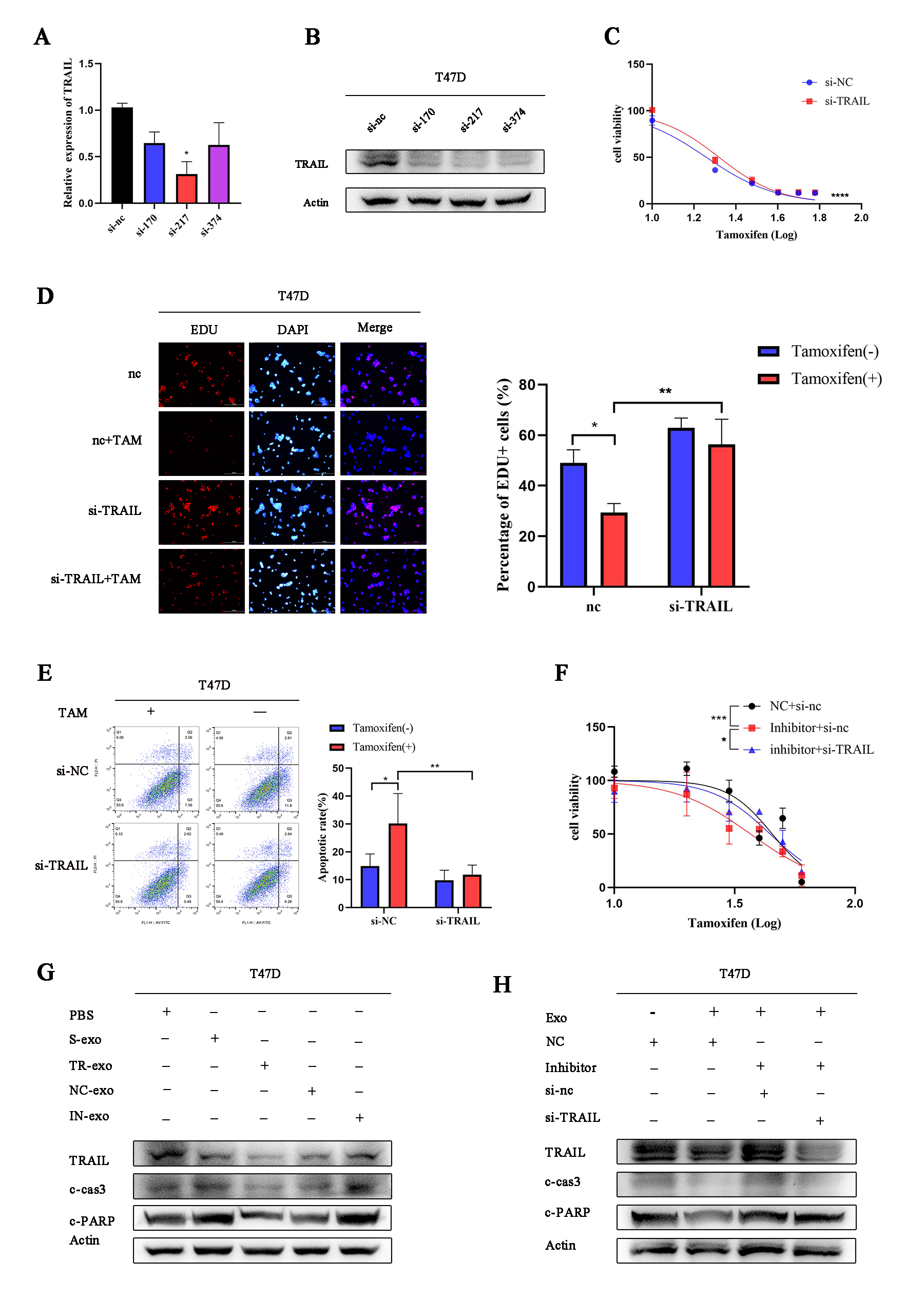

Supplement: Supplementary file 1 [file cancers-15-00899-s001.zip › Figure S3.jpg]

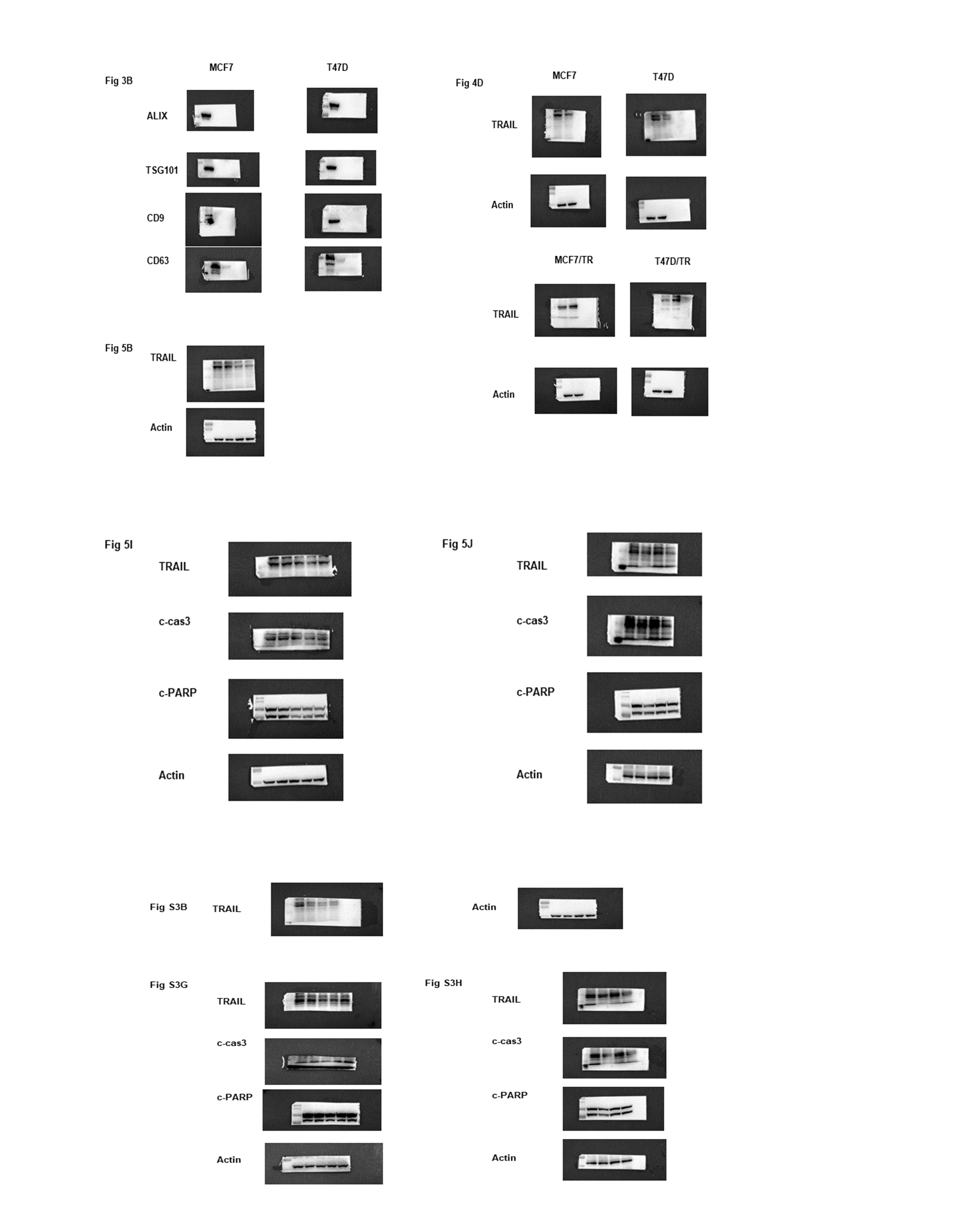

Supplement: Supplementary file 1 [file cancers-15-00899-s001.zip › Figure S4.png]
